# 754例Ⅰ期-Ⅲa期可手术切除非小细胞肺癌患者*EGFR*和*KRAS*基因突变状态及其临床意义

**DOI:** 10.3779/j.issn.1009-3419.2017.09.05

**Published:** 2017-09-20

**Authors:** 静 赵, 洁 高, 李平 郭, 晓许 胡, 琦 刘, 金银 赵, 利成 刘, 君 姜, 孟昭 王, 智勇 梁, 燕 徐, 闽江 陈, 力 张, 龙芸 李, 巍 钟

**Affiliations:** 1 100730 北京，中国医学科学院，北京协和医学院，北京协和医院呼吸科 Department of Respiratory Medicine, Peking Union Medical College Hospital, Chinese Academy of Medical Sciences and Peking Union Medical College, Beijing 100730, China; 2 100730 北京，中国医学科学院，北京协和医学院，北京协和医院病理科 Department of Pathology, Peking Union Medical College Hospital, Chinese Academy of Medical Sciences and Peking Union Medical College, Beijing 100730, China; 3 116635 大连，大连晶泰医学检验所 Dalian Gentalker Clinical Laboratory, Dalian 116635, China; 4 101300 北京，北京宏微特斯生物科技有限公司 Beijing Macro & Micro Test Biotech Company, Beijing 101300, China

**Keywords:** 肺肿瘤, *EGFR*基因, *KRAS*基因, 可手术切除标本, Lung neoplasms, Epidermal growth factor receptor gene, Kirsten rat sarcoma viral oncogene, Resectable samples

## Abstract

**背景与目的:**

表皮生长因子受体（epidermal growth factor receptor, *EGFR*）和*KRAS*基因是非小细胞肺癌（non-small cell lung cancer, NSCLC）重要的分子靶点，但目前研究主要集中在晚期NSCLC组织和血浆标本的EGFR检测，早期NSCLC组织样本中*EGFR*和*KRAS*突变特征尚不清楚。本研究将探讨Ⅰ期-Ⅲa期NSCLC *EGFR*和*KRAS*基因突变与相关临床病理特征的关系。

**方法:**

采用突变扩增系统（amplification refractory mutation system, ARMS）PCR方法检测北京协和医院病理科提供的754例Ⅰ期-Ⅲa期NSCLC组织样本的*EGFR*和*KRAS*基因突变状况，分析基因突变率及其与临床病理特征的关系。

**结果:**

*EGFR*和*KRAS*基因热点突变的突变率分别为34.5%和13.1%，其中有3例样本具有*EGFR*和*KRAS*基因的双突变。*EGFR*基因在女性中的突变率高于男性（39.5% *vs* 29.4%, *P*=0.076），在腺癌中的突变率（38.7%）高于鳞癌、腺鳞癌、大细胞癌（*P* < 0.01），但仍明显低于其他研究报道的亚裔晚期腺癌突变率（-50%）。*KRAS*基因突变在男性中的突变率高于女性（16.6% *vs* 9%, *P*=0.048），且在腺癌中的突变率也高于其他类型，但差异不显著（*P*=0.268）。与*KRAS*基因突变阳性组相比，*EGFR*基因突变阳性组在年龄分布上有年轻化的趋势（*P*=0.031, 5），在性别分布上有显著性差异（*P* < 0.01）。

**结论:**

Ⅰ期-Ⅲa期NSCLC *EGFR*基因突变率较晚期患者低，且*EGFR*和*KRAS*基因双突变的发生率为0.9%。

## Abstract (en)

**Background and objective:**

Epidermal growth factor receptor (*EGFR*) and *KRAS* gene are important driver genes of non-small cell lung cancer (NSCLC). The studies are mainly focused on detection of EGFR gene for advanced NSCLC, and the mutation feature of *EGFR* and *KRAS* gene in early NSCLC tissue is unknown. This study aims to investigate the mutations of *EGFR* and *KRAS* gene in NSCLC, and the relationship between the genotype and clinicopathologic features.

**Methods:**

The hotspot mutations in *EGFR* and *KRAS* gene in 754 tissue samples of stage Ⅰ-Ⅲa NSCLC from Department of Pathology, Peking Union Medical College Hospital were detected by modified amplification refractory mutation system (ARMS) real-time PCR kit, and analyzed their correlation with clinical variables.

**Results:**

The hotspot mutation rates in *EGFR* and *KRAS* were 34.5% and 13.1% respectively, and there were *EGFR-KRAS* double mutations in 3 samples. The mutation rate of *EGFR* was higher in females than that in males (39.5% *vs* 29.4%, *P*=0.076), significantly increased in adenocarcinomas (38.7%) compared to that in the other forms of NSCLC (*P* < 0.01), but still lower than that reported in some Asian studies of advanced adenocarcinoma (-50%). Meanwhile, the mutation rate of *KRAS* was remarkably higher in males than that in females (16.6% *vs* 9%, *P*=0.048), increased in adenocarcinomas compared to that in the other forms of NSCLC, but the difference was not significant (*P*=0.268). Samples harbored *EGFR* mutation were younger than those harbored *KRAS* mutation (*P*=0.031, 5), and had significant difference in gender between the two groups (*P* < 0.01).

**Conclusion:**

The mutation rate of *EGFR* in stag Ⅰ-Ⅲa NSCLC patients was lower than that in advanced NSCLC patients. And the percentage of the NSCLC patients with *EGFR-KRAS* double mutations is 0.9%.

非小细胞肺癌（non-small cell lung cancer, NSCLC）发病率越来越高，已成为全球癌症死亡的首要原因^[1-3]^。近20年来，NSCLC的治疗取得了飞速发展，涌现出大量新的药物和治疗手段，如分子靶向治疗^[4-7]^、免疫治疗^[8-10]^等，使患者预后大为改善。在分子靶向治疗方面，研究最成熟的当属表皮生长因子受体（epidermal growth factor receptor, EGFR）。具有*EGFR*敏感突变的患者，使用吉非替尼、厄洛替尼和阿法替尼具有更高的客观缓解率（objective response rate, ORR）、更长的无进展生存期（progression-free survival, PFS）以及更高的生活质量。当这些一、二代EGFR酪氨酸激酶抑制剂（EGFR tyrosine kinase inhibitors, EGFR-TKIs）耐药后，若耐药机制由EGFR T790M介导，使用三代EGFR-TKIs（Osimertinib, AZD9291）亦能再次产生显著疗效。因此，对*EGFR*突变的准确检测显得尤为重要。目前研究主要集中在晚期NSCLC组织和血浆标本的EGFR检测。PIONEER研究^[11]^显示，在晚期亚裔腺癌患者*EGFR*突变率高达51.4%。在另外一项IGNITE研究，再次确认了晚期亚裔NSCLC腺癌*EGFR*突变率为49.2%，与PIONEER结果一致，非腺癌患者*EGFR*突变率也高达14.1%，与欧美人群中腺癌患者突变率相当。然而，对于真实世界中，可手术切除的Ⅰ期-Ⅲa期NSCLC组织标本的EGFR突变情况尚不清楚^[12, 13]^。

另外，并非所有具有*EGFR*敏感突变的NSCLC患者对EGFR-TKIs表现出良好疗效。*KRAS*基因编码的P21蛋白位于EGFR信号通路下游，*KRAS*基因突变会使下游信号通路配体非依赖性持续性激活，不受上游靶向药物对EGFR的影响，导致细胞持续恶性增殖。*KRAS*基因突变会使NSCLC患者对EGFR-TKIs产生耐药^[14]^，同时患者预后更差^[15]^。多数研究认为*EGFR*和*KRAS*基因突变是相互排斥的^[16-19]^，但是一些临床研究表明*KRAS*基因突变可以发生在EGFR突变型患者中，但双突变发生率低于1%。因此，同时检测EGFR和KRAS对指导NSCLC患者个体化治疗具有重要临床意义^[20]^。

*EGFR*基因突变多位于外显子18-21，其中以外显子19缺失突变和外显子21L858R错义突变最为常见，约占总突变率的90%^[21]^。*KRAS*基因最常见的突变方式为点突变，90%的*KRAS*基因突变位于外显子2的第12和13密码子位点。本研究目的旨在使用Modified AMRS PCR荧光探针法检测真实世界下，未加选择的可手术切除Ⅰ期-Ⅲa期的NSCLC患者组织标本中*EGFR*和*KRAS*基因的热点突变状态，是否存在双突变以及基因突变与临床因素的相关性。

## 1 材料与方法

### 1.1 材料

标本来自北京协和医院病理科于2013年1月-2013年12月收集NSCLC标本，标本类型仅限于手术完整切除的标本，除外电子气管镜和穿刺活检标本，去除重复标本，共纳入754例，其中男性374例，女性380例；年龄24岁-92岁，中位年龄60岁；年龄分组：40岁以下、40岁-49岁、50岁-59岁、60岁-69岁、70岁以上患者样本分别为15例（2.0%）、53例（7.0%）、282例（37.4%）、240例（31.8%）和164例（21.7%）；病理分型：腺癌623例（82.5%）、鳞癌91例（12.1%）、腺鳞癌1例（0.1%）、其他39例（5.3%）。所有标本均经10%中性福尔马林固定、石蜡包埋、切片及HE染色，由病理医师复阅切片明确诊断，并确定有足够的肿瘤细胞再行后续检测。

### 1.2 方法

#### 1.2.1 DNA提取用

无菌刀片刮取5张-10张4 μm厚的连续石蜡切片中癌组织区域，保证肿瘤细胞含量在30%以上。石蜡组织脱蜡后用石蜡包埋组织DNA快速提取试剂盒（天根生化科技（北京）有限公司）提取基因组DNA。用NanoDrop 2000荧光分光光度计检测DNA的质量和浓度，所有DNA吸光度值A_260/280_均在1.8-2.0之间，用无DNase水将DNA稀释至0.5 ng/μL-10 ng/μL备用。

#### 1.2.2 突变扩增系统（amplification refractory mutation system, ARMS）

荧光PCR检测以所提取石蜡样本DNA为模板，按照*EGFR*基因和*KRAS*基因突变检测试剂盒（购自于北京华大吉比爱生物技术有限公司）操作说明书步骤，应用实时荧光定量PCR仪（ABI 7300, USA）检测*EGFR*和*KRAS*基因的常见突变，所检突变包括*EGFR*基因L858R和exon 19del突变及*KRAS*基因12和13密码子突变。检测结果由两位专业的研究人员判读和分析。如果判读结果不一致，再由第三位研究员分析得出最终决定。按照双盲试验的设计原则，试验数据的记录和获得由不同的研究员独立进行。研究员对患者组织检测结果并不知晓直到统计分析。

#### 1.2.3 测序分析

若检出*EGFR*和*KRAS*双突变的标本，采用直接测序法进行验证。针对*EGFR*基因19、21号外显子和*KRAS*基因第2号外显子设计扩增引物且在扩增引物的5’端加上M13测序引物，其中*EGFR*基因第19号外显子上下游引物分别为：5’-TGTAAAACGACGGCCAGTAATTCCCGTCGCTATC-3’；5’-GGAAACAGCTATGACCGTGGGCCTGAGGTTCAGAG-3’，*EGFR*基因第21号外显子上下游引物分别为：5’-TGTAAAACGACGGCCAGTTACTTGGAGGACCGTCGCTT-3’；5’-CAGGAAACAGCTATGACCGCTGACCTAAAGCCACCTCC-3’，*KRAS*基因第2号外显子上下游引物分别为：5’-TGTAAAACGACGGCCAGTCGATACACGTCTGCAGTCAACT-3’；5’-CAGGAAACAGCTATGACCGCATATTACTGGTGCAGGACC-3’。以所提取石蜡样本DNA为模板进行PCR扩增，反应条件：95 ℃预变性7 min；95 ℃变性30 s，56 ℃ 30 s，72 ℃延伸45 s，40个循环，最后72 ℃延伸10 min。将所得PCR产物送上海生工生物公司进行测序，使用DNAStar软件对测序结果进行比对和分析。

### 1.3 统计学分析

应用SPSS 18.0软件进行统计学分析。针对连续变量采用独立样本*t*检验，针对计数变量采用卡方检验，*P* < 0.05为差异有统计学意义。

## 2 结果

### 2.1 样本中*EGFR*、*KRAS*突变情况

本次检测涉及754例样本，共260例检出*EGFR*突变（34.5%），其中，外显子21 L858R突变124例（16.4%），外显子19缺失突变（exon 19del）142例（18.8%），有6例（0.8%）样本同时发生了L858R和19del突变。有320例样本同时进行了*KRAS*基因突变检测，共检出42例*KRAS*突变（13.1%），且全部为12号密码子突变，未检出13号密码子突变。有3例（0.9%）样本出现双突变，其中，2例为*EGFR*（L858R）和*KRAS*（G12）双突变，1例为*EGFR*（Exon 19del）和*KRAS*（G12）突变，将此3例双突变样本使用常规测序验证，结果如图 1。

**1:**
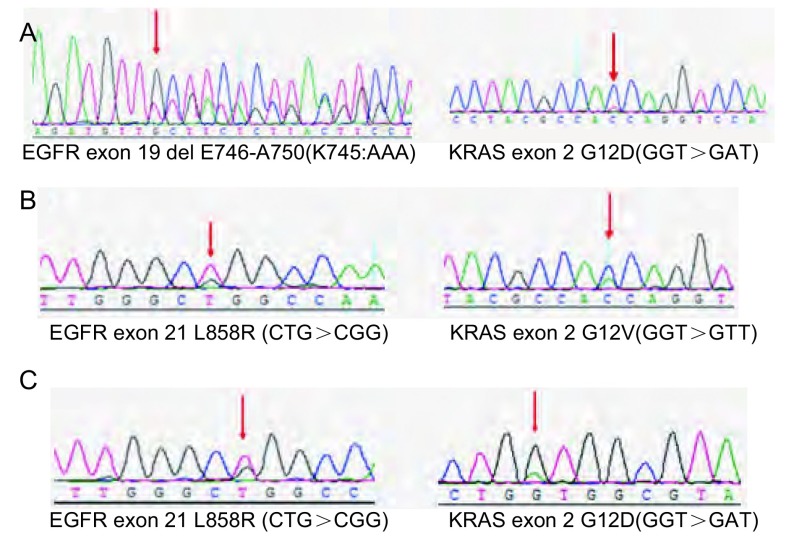
3例*EGFR-KRAS*双突变样本的DNA测序分析 DNA sequencing data of 3 specimens with *EGFR-KRAS* double mutations. EGFR: epidermal growth factor receptor.

### 2.2 *EGFR*和*KRAS*基因突变与NSCLC临床病理特征的关系

在754例NSCLC样本中，女性*EGFR*基因突变率（39.5%, 150/380）高于男性（29.4%, 110/374），但二者没有显著性差异（*P*=0.076）。60岁以下患者和60岁以上患者*EGFR*基因突变率相当[36.3%（127/350）*vs* 32.9%（133/404）]。腺癌（38.7%, 241/623）中*EGFR*基因突变率明显高于鳞癌（10.0%, 9/91）和其他类型癌（25.6%, 10/39）的突变率。在其中320例NSCLC样本中，男性*KRAS*基因突变率（16.6%, 29/175）高于女性（9.0%, 13/145），且二者具有显著差异（*P*=0.048）。60岁以下患者和60岁以上患者*KRAS*基因突变率相当[17.1%（28/164）*vs* 15.4%（24/156）]。腺癌（14.1%, 36/255）中*KRAS*基因突变率高于鳞癌（7.7%, 2/26）和其他类型癌（10.5%, 4/38），详见表 1。

**1:** *EGFR*及*KRAS*基因突变情况与患者临床病理特征之间的关系 The relationship between *EGFR* and *KRAS* mutations and clinical variables of patients

Clinical variables	*n*	*EGFR*		*P*	*n*	*KRAS*		*P*
		Mutant	Wild-type			Mutant	Wild-type	
Gender				0.076				0.048
Male	374	110 (29.4)	264 (70.6）		175	29 (16.6)	146 (83.4)	
Female	380	150 (39.5)	230 (60.5)		145	13 (9.0)	132 (91.0)	
Age (years)				0.357				0.762
< 60	350	127 (36.3)	223 (63.7)		164	28 (17.1)	136 (82.9)	
≥60	404	133 (32.9)	271 (67.1)		156	24 (15.4)	132 (84.6)	
Histology				< 0.01				0.268
Adenocarcinoma	623	241 (38.7)	382 (61.3)		255	36 (14.1)	219 (85.9)	
Squamous	91	9 (10.0)	82 (90.0)		26	2 (7.7)	24 (92.3)	
Adenosquamous	1	0 (0.0)	1 (100.0)		1	0 (0.0)	1 (100.0)	
Others^*^	39	10 (25.6)	29 (74.4)		38	4 (10.5)	34 (89.5)	
^*^：Others include large cell carcinoma and sarcomatoid carcinoma.								

### 2.3 基于*EGFR*和*KRAS*基因分型的单因素分析

在*EGFR*或*KRAS*基因突变阳性的样本中，EGFR阳性组的中位初诊年龄为58岁，平均年龄为（58.6±10.1）岁，KRAS阳性组的中位初诊年龄为61.5岁，平均年龄为（62.4±8.4）岁，*EGFR*阳性组患者年龄分布较KRAS阳性组具有年轻化趋势（*P*=0.031, 5）。在性别分布方面，KRAS阳性组的男性分布比例明显高于EGFR组（69% *vs* 42.3%, *P* < 0.01）。在病理类型分布方面，两组均以腺癌为主，并未发现有显著差异（*P*=0.228）（图 2）。

**2:**
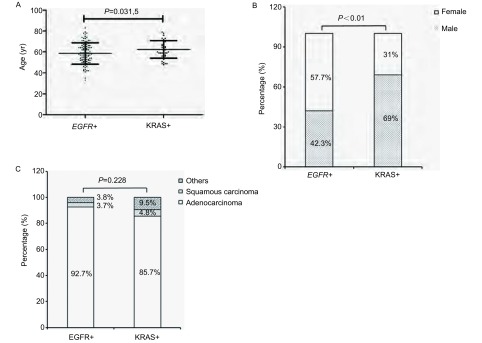
基于*EGFR*和*KRAS*基因分型的单因素分析。A:基于*EGFR*和*KRAS*分型的年龄分布情况；B：基于*EGFR*和*KRAS*分型的性别分布情况；C：基于*EGFR*和KRAS分型的病理类型分布情况。 Single factor analysis based on *EGFR* and *KRAS* genotype. A: Age distribution based on *EGFR* and *KRAS* genotype; B: Gender distribution based on *EGFR* and *KRAS* genotype; C: Pathological type based on *EGFR* and *KRAS* genotype.

## 3 讨论

本研究是一项专门针对Ⅰ期-Ⅲa期可手术切除NSCLC患者*EGFR*和*KRAS*基因突变状况的调查研究。研究显示，在未加选择可切除的Ⅰ期-Ⅲa期NSCLC患者，*EGFR*基因突变率为34.5%，这与早期文献^[22, 23]^所报道的一致。但对于腺癌，此研究*EGFR*突变率仅为38.7%，明显低于PIONEER和IGNITE研究所报道的50%左右^[11]^。在PIONEER和IGNITE研究中，均是纳入的Ⅳ期腺癌患者，而我们的标本为Ⅰ期-Ⅲa期，这种早晚期标本差异可能是导致突变率不同的一个重要原因。在一项EGFR-TKIs术后辅助治疗的随机Ⅲ期临床研究中（BR.19研究）^[20]^，研究者亦发现手术标本中*EGFR*的突变率明显低于晚期患者，仅3%（15/503），而且分期越早的标本，其*EGFR*突变率越低，这些发现似乎也在一定程度上支持我们的结论。这也反映早期NSCLC和晚期NSCLC可能具有不同的生物学行为，*EGFR*基因在其中所起的作用不同，因此制定综合治疗策略需考虑这种差异^[24, 25]^。

既往研究^[26, 27]^表明，*EGFR*突变与患者性别、种族、吸烟、病理类型有关，其在亚裔、女性、不吸烟、腺癌的患者中发生率较高。本研究在754例Ⅰ期-Ⅲa期NSCLC样本中也发现，*EGFR*基因突变率在腺癌中较高，同时女性较男性也有升高趋势（39.5% *vs* 29.4%, *P*=0.076），这与既往研究结果一致。

*KRAS*基因是EGFR信号转导通路下游的重要节点，该基因的突变可以导致EGFR-TKI原发性耐药，同时它亦是判断NSCLC患者预后的重要指标。*KRAS*基因突变具有明显的地域差异，西方人群中*KRAS*突变率约25%，明显高于东亚人群10%，且*KRAS*基因突变多见于男性，吸烟以及肺腺癌^[27]^。在本研究中，*KRAS*基因的突变率为13.1%，且腺癌的突变率（14.1%）高于鳞癌（7.7%）和其他类型癌（10.5%）。这些发现也与既往研究^[27, 28]^一致。

在NSCLC中同时存在*EGFR*和*KRAS*突变非常罕见，一直以来研究者认为两者互斥^[16-18]^。但在本研究中，共有3例样本在*EGFR*和*KRAS*基因上同时发生了突变，均为女性肺腺癌患者，且经直接测序法验证，说明*EGFR*和*KRAS*双突变可以同时存在，*KRAS*的突变可能会导致具有*EGFR*敏感突变患者对EGFR-TKIs耐药。在临床中，约30% EGFR阳性NSCLC患者对EGFR-TKIs靶向治疗无应答，即发生原发性耐药^[29]^。本研究，在320例检测*EGFR*和*KRAS*突变的样本中，*EGFR*的突变率为30.3%（97/320），其中在*EGFR*突变阳性样本中，*KRAS*的突变率为3.1%（3/97），其远远低于30%，说明EGFR-TKIs原发耐药除了*KRAS*突变外，还有很多其他机制参与，如BIM表达水平等^[29]^。

总之，本研究提示在Ⅰ期-Ⅲa期可手术的NSCLC样本中*EGFR*基因突变率低，*EGFR*基因突变和*KRAS*基因突变可同时存在。但该研究仍有较多缺陷，它是一项回顾性研究，所得结论是与其他研究结果进行比较得出，未纳入Ⅳ期标本做为对照，部分临床资料收集不完善以及EGFR检测内容不全，这些均需要在后续研究中改进和完善，并进一步验证研究结论的可靠性。
